# The Role of Gut Microbiota and the Potential Effects of Probiotics in Heart Failure

**DOI:** 10.3390/medicina60020271

**Published:** 2024-02-04

**Authors:** Carmine Petruzziello, Angela Saviano, Luca Luigi Manetti, Noemi Macerola, Veronica Ojetti

**Affiliations:** 1 Emergency Department, Ospedale San Carlo di Nancy—GVM Care & Research, 00165 Rome, Italy; cpetruzziello@gvmnet.it (C.P.); llmanetti@gvmnet.it (L.L.M.); 2 Emergency Department, Policlinico Universitario A. Gemelli IRCCS, 00168 Rome, Italy; angela.saviano@policlinicogemelli.it; 3 Internal Medicine, Ospedale San Carlo di Nancy—GVM Care & Research, 00165 Rome, Italy; nmacerola@gvmnet.it; 4 Deaprtment of Internal Medicine, Università Cattolica del Sacro Cuore, 00168 Rome, Italy

**Keywords:** heart failure, probiotics, gut microbiota, dysbiosis, gut–heart axis

## Abstract

Heart failure (HF) remains a significant global health challenge, affecting millions of individuals worldwide and posing a substantial burden on healthcare systems. HF is a syndrome of intricate pathophysiology, involving systemic inflammation, oxidative stress, metabolic perturbations, and maladaptive structural changes in the heart. It is influenced by complex interactions between cardiac function, systemic physiology, and environmental factors. Among these factors, the gut microbiota has emerged as a novel and intriguing player in the landscape of HF pathophysiology. The gut microbiota, beyond its role in digestion and nutrient absorption, impacts immune responses, metabolic processes, and, as suggested by evidence in the literature, the development and progression of HF. There is a bidirectional communication between the gut and the heart, often known as the gut–heart axis, through which gut microbiota-derived metabolites, immune signals, and microbial products exert profound effects on cardiovascular health. This review aims to provide a comprehensive overview of the intricate relationship between the gut microbiota and HF. Additionally, we explore the potential of using probiotics as a therapeutic strategy to modulate the gut microbiota’s composition and attenuate the adverse effects observed in HF. Conventional therapeutic approaches targeting hemodynamic and neurohormonal dysregulation have substantially improved the management of HF, but emerging research is exploring the potential implications of harnessing the gut microbiota for innovative approaches in HF treatment.

## 1. Introduction

Heart failure (HF) remains a significant and escalating global health challenge, affecting millions of individuals worldwide and posing a substantial burden on healthcare systems [1]. This complex cardiovascular syndrome arises from various etiologies, leading to impaired cardiac function and the inability of the heart to effectively pump blood to meet the body’s metabolic demands. Despite advances in medical, surgical, and interventional therapies, the morbidity, mortality, and economic burden associated with HF continue to be considerable [2].

Conventional therapeutic approaches targeting hemodynamic and neurohormonal dysregulation have substantially improved the management of HF [3]. These approaches encompass the use of medications such as angiotensin-converting enzyme inhibitors, beta-blockers, and mineralocorticoid receptor antagonists, along with device-based interventions like implantable cardioverter–defibrillators and cardiac resynchronization therapy [4]. However, HF remains a syndrome of intricate pathophysiology, involving systemic inflammation, oxidative stress, metabolic perturbations, and maladaptive structural changes in the heart [5].

Emerging research is broadening our understanding of the factors contributing to HF’s development and progression. It is increasingly recognized that HF is not solely a disorder of the heart but is influenced by complex interactions between cardiac function, systemic physiology, and environmental factors. Among these factors, the gut microbiota has emerged as a novel and intriguing player in the landscape of HF pathophysiology.

The gut microbiota, a diverse community of microorganisms residing in the gastrointestinal tract, has gained prominence for its potential role in influencing human systemic health [6]. Beyond its role in digestion and nutrient absorption, the gut microbiota profoundly impacts immune responses, metabolic processes, and even neurological functions [7]. 

Recent studies have illuminated the bidirectional relationship between gut microbiota composition and cardiac function, shedding light on how alterations in the gut microbial ecosystem can contribute to the development and progression of HF and its complications [8]. Understanding this intricate crosstalk between the gut and the heart holds promise for novel therapeutic strategies in HF management.

This connection is rooted in the bidirectional communication between the gut and the heart, often referred to as the gut–heart axis, through which gut microbiota-derived metabolites, immune signals, and microbial products can exert profound effects on cardiovascular health [9].

This review aims to provide a comprehensive overview of the intricate relationship between the gut microbiota and HF. We delve into the mechanisms by which alterations in gut microbiota composition might contribute to systemic inflammation, oxidative stress, and metabolic dysregulation, all of which play pivotal roles in HF progression. Additionally, we explore the potential of using probiotics as a therapeutic strategy to modulate gut microbiota composition and attenuate the adverse effects observed in HF. Through an analysis of preclinical and clinical evidence, we evaluate the efficacy and safety of probiotic interventions in HF management. Moreover, we discuss the challenges, future directions, and potential implications of harnessing the gut microbiota for innovative approaches in HF treatment.

## 2. Materials and Methods

This narrative review includes studies published in English over the last 25 years on the topics of microbiota and heart failure, focusing on the circulating system and the gastrointestinal tracts. We searched on PubMed^®^, UptoDate^®^, and Cochrane^®^. The keywords we searched for were “heart failure OR HF AND microbiota OR probiotics” in the titles of articles. The inclusion and exclusion criteria are reported in Table 1. After the application of the inclusion and exclusion criteria, we were able to select 31 articles. All the articles have been read, and their bibliographies have been checked in order to select other works reputed to be relevant based on the opinions of the authors. No ethical approval was required to perform this review.

## 3. Gut Microbiota Dysbiosis in Heart Failure

The human gut harbors a complex and diverse microbial ecosystem known as the gut microbiota, which encompasses bacteria, viruses, fungi, archaea, and other microorganisms [10]. 

When in balance, this intricate microbial community plays a pivotal role in various physiological processes, including nutrient metabolism, energy extraction, immune modulation, and even neurological functions [7]. 

The normal composition of the gut microbiota in healthy subjects consists mainly of two phyla, Firmicutes and Bacteroidetes, followed by Proteobacteria, Actinobacteriota and Verrucomicrobiota [11]. 

Alterations in gut microbiota composition have been observed among individuals with HF, especially in the decompensated phase, strengthening the connection between gut dysbiosis and HF pathogenesis. In particular, a reduction in microbial diversity has been demonstrated, with an expansion in bacteria belonging to the phylum Proteobacteria (mainly pathogenic bacteria) and a decrease in beneficial bacteria [11,12,13]. Several research studies have demonstrated that HF patients show a higher abundance of pathogenic bacteria, including but not limited to *Salmonella*, *Shigella*, *Escherichia*, *Campylobacter*, *Klebsiella*, *Yersinia*, and *Clostridium* [14,15]. Some of these bacteria have been associated with the severity of the disease as expressed by the New York Heart Association (NYHA) functional classes [16]. With respect to controls, HF patients are also characterized by less abundance of short-chain fatty acid (SCFA)-producing bacteria such as *Blautia*, *Erysipelotrichaceae*, *Collinsella*, *Ruminococcaceae*, *Lachnospiraceae*, *Faecalibacterium*, *Eubacterium rectale*, and *Dorea Longicatena* [17,18,19].

Inflammation is a central player in the progression of HF, contributing to cardiac remodeling and dysfunction [20]. One of the most relevant aspects in gut-derived inflammation is the gut’s altered circulation as a result of reduced cardiac output. The consequent sympathetic vasoconstriction leads to a redistribution of intestinal blood flow, with relative ischemic phenomena at the villus tip, venous congestion, and bowel wall edema [21]. This edematous decompensation promotes the production of pro-inflammatory cytokines, with a significant shift of the gut flora into pathogenetic phyla and an increase in intestinal permeability, with the subsequent translocation into the bloodstream of gram-negative intestinal bacteria and endotoxins.

**Figure 1:**
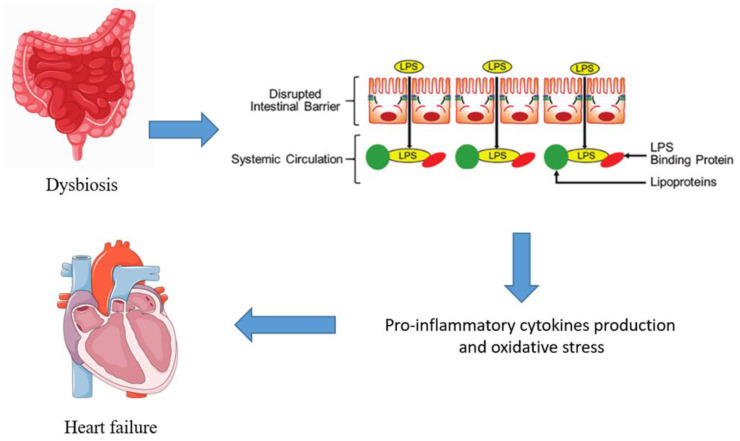
Pathophysiological pathways between gut microbiota and heart failure.

Among them, lipopolysaccharides’ (LPS) translocation into systemic circulation represents one of the most relevant effects of dysbiosis [22]. Elevated levels of LPS can trigger immune responses by interacting with the host mucosal surface cells through the Toll-like Receptor 4 (TLR4) and nucleotide oligomerization domain-containing receptors [23], thus contributing to a state of endotoxemia and systemic inflammation. Additionally, the gut microbiota’s role in immune system modulation can lead to the production of proinflammatory cytokines, further exacerbating the inflammatory milieu observed in heart failure [24]. Thus, dysbiosis-driven inflammation within the gut has the potential to fuel the systemic inflammatory state observed in HF (Figure 1).

Oxidative stress, characterized by an imbalance between reactive oxygen species (ROS) production and antioxidant defenses, is a hallmark of HF pathophysiology [25]. Dysbiosis can influence oxidative stress through multiple mechanisms. For instance, microbial-derived metabolite Trimethylamine N-oxide (TMAO), produced from the metabolism of dietary components choline and carnitine, has been associated with endothelial dysfunction and oxidative stress [8]. Moreover, a dysbiosis-induced reduction in short-chain fatty acids (SCFAs) could contribute to the redox imbalance observed in HF by disrupting the integrity of intestinal barrier function [26]. A large body of evidence suggests the pivotal role of oxidative stress and LPS-derived endotoxemia in metabolic disturbances [27]. Dysbiosis-induced changes in gut microbiota composition can influence metabolic pathways and contribute to the metabolic dysregulation observed in HF. The gut microbiota plays a critical role in nutrient absorption and metabolism, and dysbiosis can disrupt these processes, impacting energy extraction and utilization [28]. Furthermore, dysbiosis can influence gut barrier function, potentially leading to the translocation of microbial components into systemic circulation [29]. This process, known as metabolic endotoxemia, has been associated with insulin resistance and metabolic dysfunction [30]. Therefore, dysbiosis-induced metabolic disturbances within the gut have the potential to contribute to the systemic metabolic dysregulation observed in HF.

The gut–heart axis represents a dynamic and intricate network of communication between the gut microbiota’s composition and cardiovascular health. This multifaceted relationship underscores the far-reaching impact of the gut on the heart, with profound implications for understanding HF pathophysiology. Dysbiosis represents the primum movens of a cascade of events reverberating throughout the cardiovascular system [31], recognizing as one of the key components the induction of inflammation and oxidative stress. Inflammatory processes within the gut, driven by alterations in microbiota composition, can lead to the production of proinflammatory cytokines and other mediators that find their way into the systemic circulation. These circulating inflammatory signals can contribute to endothelial dysfunction, a key initiating event in the development of atherosclerosis and cardiac dysfunction. In fact, the low–medium systemic inflammatory grade seen in HF patients has deleterious effects on several cell types, included endothelial cells.

Several pro-inflammatory mediators, such as IL-1β, IL-6, TNF-α, adhesion molecules, and immune cells, can induce fibrosis and influence cardiac function and are correlated with a worse prognosis [32]. Furthermore, low-grade LPS endotoxemia was observed in the acute phase of myocardial infarction; the immunochemistry analysis of coronary thrombi demonstrated the role of the LPS-TLR4 axis in atherothrombosis, revealing an interplay which might also contribute to thrombosis via the release of neutrophil extracellular traps (NETs) and ROS formation [33].

The gut, once viewed as a distant organ, is now recognized as a significant source of inflammatory stimuli that can exert a profound impact on the vascular endothelium and the heart itself. Similarly, oxidative stress, resulting from an imbalance between the production of ROS and antioxidant defenses, can be exacerbated by gut dysbiosis. Dysbiotic gut microbiota may produce ROS or modulate host antioxidant systems, disturbing the delicate equilibrium within the cardiovascular system. This oxidative imbalance can further contribute to endothelial dysfunction, cardiac remodeling, and ultimately, heart failure progression [26]. The gut microbiota’s influence extends beyond inflammation and oxidative stress, encompassing an impact on immune responses and metabolic pathways [34]. Dysbiotic gut microbiota can disrupt immune homeostasis, altering the balance between proinflammatory and anti-inflammatory signals. This immune dysregulation can have profound consequences for the cardiac remodeling process in heart failure. Inflammation and immune activation are integral components of cardiac remodeling, a complex phenomenon that involves structural and functional changes in the heart. Dysregulated immune responses can contribute to adverse remodeling, promoting fibrosis, hypertrophy, and ventricular dysfunction. The gut microbiota’s influence on immune responses, including the modulation of immune cell populations and cytokine profiles, can thereby shape the cardiac remodeling trajectory in heart failure patients.

The emerging understanding of the link between gut microbiota dysbiosis and HF pathogenesis holds clinical implications for HF management. Strategies aimed at modulating gut microbiota composition, such as probiotic interventions, offer a promising avenue for intervention. By targeting dysbiosis-induced inflammation, oxidative stress, and metabolic disturbances, probiotics have the potential to influence pathways critical to HF progression [35]. However, challenges remain in identifying the optimal probiotic strains, dosages, and treatment durations for HF management. Moreover, individual variability in gut microbiota composition and responses to probiotics introduces complexity in translating preclinical and clinical findings into effective therapeutic strategies [36].

## 4. Probiotics as Modulators of Gut Microbiota

Probiotics, coined as “beneficial” or “friendly” bacteria, have gained substantial attention for their potential to confer health benefits on the host [10]. These living microorganisms, when administered in appropriate quantities, can exert positive effects on various physiological processes. In the context of heart failure, the use of probiotics as a therapeutic strategy has emerged as a compelling avenue due to their capacity to modulate gut microbiota composition and potentially influence heart failure-related outcomes.

Probiotics exert their effects through diverse mechanisms that impact gut microbiota composition and host physiology. One key mechanism is their ability to compete with pathogenic microorganisms for resources and adhesion sites within the gut, thereby promoting a healthier microbial balance [37]. Additionally, probiotics can enhance gut barrier function by strengthening the integrity of tight junctions between intestinal cells [38]. This barrier-enhancing effect has the potential to mitigate the translocation of microbial components into systemic circulation, which is implicated in heart failure-related inflammation and oxidative stress [39].

Another notable mechanism is the modulation of immune responses [40]. Probiotics can regulate the balance between proinflammatory and anti-inflammatory cytokines, thus influencing the systemic inflammatory state observed in heart failure [41]. Moreover, probiotics can impact microbial metabolites such as SCFAs, which have immunomodulatory effects and can contribute to maintaining gut health [42]. By influencing these pathways, probiotics can potentially attenuate heart failure-associated systemic inflammation.

Preclinical studies have provided valuable insights into the potential benefits of probiotics in heart failure management. Animal models have demonstrated that probiotic supplementation can lead to improvements in cardiac structure and function [43]. These benefits include reductions in left ventricular hypertrophy, attenuation of fibrosis, and enhancements in cardiac contractility. Mechanistically, animal studies have suggested that probiotics’ effects on inflammation, oxidative stress, and gut barrier function contribute to their beneficial impact on heart failure-related outcomes [9].

Clinical investigations have explored the effects of probiotic supplementation in heart failure patients [39]. These studies have reported enhancements in exercise tolerance, quality of life, and biomarkers of inflammation and oxidative stress [36,44]. Moreover, specific probiotic strains led to reductions in inflammatory markers and improvements in gut microbiota composition. Similar findings were reported in a study by Zhang et al., which observed improvements in metabolic profiles and inflammatory markers with probiotic supplementation [45]. However, it is important to acknowledge that the optimal strains, dosages, and treatment durations of probiotics for heart failure management remain areas necessitating further exploration [5]. Moreover, individual variability in gut microbiota composition and responses to probiotics introduces a layer of complexity to the interpretation of clinical outcomes [46].

## 5. Preclinical and Clinical Evidence

Preclinical models have provided invaluable insights into the potential benefits of probiotics [47], also in the context of heart failure [47,48]. Animal studies have demonstrated that probiotic supplementation can lead to improvements in cardiac structure and function. These benefits include reductions in left ventricular hypertrophy, attenuation of fibrosis, and enhancements in cardiac contractility. Animal models have also contributed to deciphering the potential mechanisms underlying these effects, encompassing the modulation of inflammation and oxidative stress pathways [49].

Preclinical models, particularly animal studies, have played a pivotal role in unraveling the intricate mechanisms through which probiotics exert their favorable effects in the context of heart failure [50]. These models provide a controlled environment to investigate the impact of specific probiotic strains on various aspects of cardiac function and pathophysiology. Studies using animal models have unveiled the remarkable ability of certain probiotic strains to modulate inflammatory responses within the cardiovascular system [50]. Inflammation is a central player in heart failure pathogenesis, contributing to cardiac remodeling and dysfunction [20]. Preclinical research has shown that specific probiotics can effectively reduce the production of proinflammatory cytokines and dampen the activation of proinflammatory pathways. By mitigating excessive inflammation, probiotics act as regulators of the inflammatory milieu that often characterizes HF. This anti-inflammatory action can help attenuate the adverse cardiac remodeling processes that lead to structural changes in the heart, thereby preserving cardiac function and overall cardiovascular health. Given the critical role of oxidative stress in HF pathophysiology, preclinical studies have illuminated how specific probiotic strains can enhance antioxidant defenses within the cardiovascular system. This enhancement is vital in mitigating oxidative stress and its detrimental impact on cardiac tissues [51]. By bolstering antioxidant mechanisms, probiotics help to restore the delicate balance between the production of reactive oxygen species (ROS) and the body’s ability to neutralize them. This restoration can prevent oxidative stress-induced damage to cardiac cells and maintain the structural and functional integrity of the heart. These findings underscore the multifaceted approach by which probiotics can counteract key pathological processes in heart failure. By concurrently addressing inflammation and oxidative stress, probiotics offer a comprehensive strategy to mitigate the underlying drivers of cardiac dysfunction.

In preclinical models, probiotics have demonstrated their potential to modulate the inflammatory milieu, reduce the production of proinflammatory mediators, and enhance antioxidant defenses. These actions collectively contribute to the preservation of cardiac structure and function, providing a foundation for their consideration as adjunctive therapies in heart failure management.

The insights gained from preclinical models not only shed light on the mechanisms of probiotic action but also lay the groundwork for further exploration in clinical settings. These studies serve as a bridge between laboratory discoveries and potential clinical applications, ultimately advancing our understanding of probiotics’ therapeutic potential in the context of heart failure.

Clinical studies that investigate the impact of probiotics on heart failure patients are summarized in Table 2.

These trials have reported enhancements in exercise tolerance, quality of life, and biomarkers of inflammation and oxidative stress [55]. For instance, Awoyemi A. et al. explored the effects of probiotics on gut microbiota in heart failure patients [52]. The study found that specific probiotic strains led to an improvement in gut microbiota composition, as well as an improvement in the left ventricular ejection fraction. Similarly, Moludi J. et al. conducted a trial that observed improvements in metabolic profiles and inflammatory markers with probiotic supplementation [53]. These clinical findings align with the mechanistic insights garnered from preclinical models, underscoring the potential translational relevance of probiotics in heart failure management.

While the evidence from preclinical models and clinical trials is promising, several considerations and limitations warrant attention. Variability in study designs, probiotic strains, dosages, and treatment durations can influence outcomes, complicating direct comparisons between studies. Moreover, individual patient characteristics, including gut microbiota composition, may contribute to variable responses to probiotic interventions [57]. These factors highlight the need for personalized approaches and the identification of biomarkers that can predict individual responses to probiotics.

Additionally, the long-term safety of probiotics and their potential interactions with conventional heart failure therapies require careful evaluation [58]. Heart failure patients are often on multiple medications, and potential interactions between probiotics and these medications must be thoroughly investigated to ensure patient safety and optimize therapeutic outcomes.

## 6. Challenges and Future Directions

While the potential of probiotics in heart failure management is promising, several challenges and areas for future exploration must be addressed to optimize their therapeutic utility.

One of the challenges lies in the strain-specific effects of probiotics. Different probiotic strains can exert distinct effects on gut microbiota composition and host physiology [50]. Therefore, identifying the most suitable strains for heart failure management is essential. Moreover, individual variability in gut microbiota composition presents a hurdle in predicting patient responses to probiotics [57]. Personalized approaches that consider patients’ baseline microbiota and tailor probiotic interventions accordingly could enhance therapeutic efficacy.

While preclinical models and clinical trials have provided insights into the mechanisms through which probiotics exert their effects, further mechanistic elucidation is required. A deeper understanding of the molecular pathways influenced by probiotics can guide the development of targeted interventions [59]. For instance, uncovering the interplay between probiotics, gut metabolites, immune responses, and cardiac function could inform the design of more refined and effective probiotic-based strategies.

The diversity of probiotic formulations and dosages further adds complexity to their clinical application [39]. Identifying the optimal combination of probiotic strains and dosages that can achieve sustained and beneficial effects is essential for translating research findings into effective interventions [60]. Additionally, considerations of stability, viability, and delivery mechanisms are crucial to ensuring that probiotics reach the gut in active forms.

The long-term safety of probiotic interventions, particularly in the context of chronic conditions like heart failure, requires thorough investigation [58]. Heart failure patients often receive multiple medications, and the potential interactions between probiotics and these medications need to be carefully assessed to prevent adverse effects or reduced therapeutic efficacy [53].

Translating the promising findings from preclinical models and clinical trials into routine clinical practice poses a substantial challenge [61]. Implementation strategies, healthcare provider education, and patient adherence to probiotic regimens are critical factors influencing the successful integration of probiotics into heart failure management. Clear guidelines and recommendations for probiotic use in heart failure patients are essential to bridge the gap between research and clinical application.

The emerging field of gut microbiota research offers exciting avenues for advancing heart failure management. Innovative approaches, such as precision medicine and microbiota-targeted therapies, hold the potential to revolutionize how heart failure is treated [62]. Multi-omics analyses, including metagenomics and metabolomics, can provide deeper insights into the gut–heart axis and guide the development of more tailored interventions [63]. Additionally, collaborative efforts between researchers, clinicians, and industry partners are essential to accelerate the translation of scientific discoveries into effective therapies for heart failure patients.

## 7. Conclusions and Implication

The intricate interplay between the gut microbiota and heart failure presents a compelling opportunity for innovative therapeutic interventions. The emerging field of gut–heart axis research has illuminated the potential of probiotics as modulators of gut microbiota composition, with the capacity to impact inflammation, oxidative stress, and metabolic dysregulation—all of which contribute to heart failure progression. The evidence from preclinical models and clinical trials underscores the potential benefits of probiotic supplementation in heart failure management.

While the evidence supporting probiotics as potential adjunctive therapies in heart failure management is promising, the journey from scientific insight to practical clinical strategies presents a multifaceted set of challenges [64]. These challenges encompass various dimensions, each of which demands careful consideration and investigation.

A fundamental challenge arises from the strain-specific effects of probiotics, emphasizing that not all probiotic strains are created equal. The intricate interplay between gut microbiota and probiotics suggests that certain strains may be more efficacious than others in modulating the gut microbiota towards a state that benefits heart failure patients. Deciphering the optimal probiotic strain or combination of strains represents a crucial step in realizing their full potential.

Moreover, the substantial variability in gut microbiota composition among individuals further complicates the translation of probiotics into clinical practice. Patients with heart failure exhibit a diverse range of microbiota profiles, necessitating personalized approaches to probiotic interventions. Tailoring probiotics based on an individual’s baseline microbiota could enhance therapeutic efficacy and minimize the risk of non-responsiveness [65].

The determination of optimal probiotic dosages poses another critical challenge. Finding the right balance between providing an effective dose and avoiding potential side effects or complications is a delicate task. The safety profiles of probiotics, particularly in the context of chronic conditions like heart failure, necessitate a thorough investigation. Ensuring that probiotic interventions do not introduce unintended risks or interact with concurrent heart failure medications is paramount.

Additionally, the assessment of long-term outcomes is crucial to establishing the sustained benefits and safety of probiotics in heart failure management. While short-term studies may offer promising insights [54,56], understanding the potential for probiotics to exert lasting effects and improve the overall prognosis of heart failure patients requires rigorous, extended investigations.

To overcome these challenges, researchers are increasingly turning to advanced technologies such as metagenomics and metabolomics. These cutting-edge approaches provide a deeper understanding of the gut–heart axis by enabling comprehensive analysis of the gut microbiota and associated metabolic processes. Metagenomics allows for the characterization of microbial communities at the genetic level, unveiling intricate details about their composition and functionality. Metabolomics, on the other hand, explores the intricate web of small molecules and metabolites generated by both the gut microbiota and the host, shedding light on the molecular mechanisms at play.

By leveraging these advanced tools, researchers can gain deeper insights into how probiotics influence the gut–heart axis, paving the way for more personalized and precise interventions. These technologies hold the potential to identify specific microbial signatures or metabolic pathways that correlate with therapeutic responses to probiotics, ultimately guiding the development of tailored probiotic regimens.

In navigating these challenges and capitalizing on advanced technologies, the translation of probiotics from promising research findings to effective clinical strategies for heart failure management becomes an exciting frontier, holding the potential to transform the landscape of heart failure care.

The field of gut microbiota research holds vast potential for advancing heart failure management and transforming patient care [66]. Further research is needed to unravel the mechanistic intricacies underlying the probiotic–gut–heart connection. Elucidating the specific pathways and interactions through which probiotics modulate gut microbiota composition and impact heart failure-related outcomes will enable the design of more targeted interventions. Additionally, clinical trials exploring the long-term safety and efficacy of probiotics, as well as their interactions with conventional heart failure therapies, are warranted.

The journey towards incorporating probiotics into heart failure management is a collaborative endeavor that involves researchers, clinicians, regulatory authorities, and industry partners [67]. Robust clinical trials, evidence-based guidelines, and well-defined strategies for probiotic implementation are needed to bridge the gap between scientific discoveries and routine clinical practice. Continued interdisciplinary collaboration will be crucial in translating the potential of probiotics into tangible benefits for heart failure patients.

## Figures and Tables

**Table 1:** List of inclusion and exclusion criteria.

Inclusion Criteria	Exclusion Criteria
Type of article: in order of importance, we have considered clinical trials, observational studies, systematic reviews, and narrative reviews.	Topic: articles regarding only the topic “microbiota” or only the topic “heart failure”; articles treating other topics.
Language: only articles written in English.	
Year of publication: articles written in the last 25 years.	

**Table 2:** Summarization of clinical studies of the use of probiotics in HF.

Authors, Year	Study Type	Probiotic Used	Conclusion of the Study
Awoyemi A et al., 2021 [52]	multicenter, prospective, randomized, open label	Saccharomyces boulardii	Improvement in left ventricular ejection fraction
Moludi J et al., 2020 [53]	single-center, double-blind placebo-controlled, randomized clinical study	Lactobacillus rhamnosus GG	Improvement in serum biomarkers, improvement in left ventricular ejection fraction
Pourrajab B et al., 2022 [54]	randomized, triple-blind clinical trial	Mix of probiotic yogurt	Improvement in serum biomarkers
Karim A et al., 2022 [55]	single-center, double-blind placebo-controlled, randomized clinical study	Mix of probiotics	Improvement in serum biomarkers
Costanza A et al., 2015 [56]	randomized, double-blind, placebo-controlled pilot trial	Saccharomyces boulardii	Improvement in serum biomarkers, improvement in left ventricular ejection fraction
